# Deferasirox-induced iron depletion promotes BclxL downregulation and death of proximal tubular cells

**DOI:** 10.1038/srep41510

**Published:** 2017-01-31

**Authors:** Diego Martin-Sanchez, Angel Gallegos-Villalobos, Miguel Fontecha-Barriuso, Susana Carrasco, Maria Dolores Sanchez-Niño, Francisco J Lopez-Hernandez, Marta Ruiz-Ortega, Jesus Egido, Alberto Ortiz, Ana Belén Sanz

**Affiliations:** 1 Research Institute-Fundacion Jimenez Diaz, Autonoma University, Madrid, Spain; 2 IRSIN, Madrid, Spain; 3 REDINREN, Madrid, Spain; 4 Biomedical Research Institute of Salamanca, University of Salamanca, Salamanca, Spain

## Abstract

Iron deficiency has been associated with kidney injury. Deferasirox is an oral iron chelator used to treat blood transfusion-related iron overload. Nephrotoxicity is the most serious and common adverse effect of deferasirox and may present as an acute or chronic kidney disease. However, scarce data are available on the molecular mechanisms of nephrotoxicity. We explored the therapeutic modulation of deferasirox-induced proximal tubular cell death in culture. Deferasirox induced dose-dependent tubular cell death and AnexxinV/7AAD staining showed features of apoptosis and necrosis. However, despite inhibiting caspase-3 activation, the pan-caspase inhibitor zVAD-fmk failed to prevent deferasirox-induced cell death. Moreover, zVAD increased deferasirox-induced cell death, a feature sometimes found in necroptosis. Electron microscopy identified mitochondrial injury and features of necrosis. However, neither necrostatin-1 nor RIP3 knockdown prevented deferasirox-induced cell death. Deferasirox caused BclxL depletion and BclxL overexpression was protective. Preventing iron depletion protected from BclxL downregulation and deferasirox cytotoxicity. In conclusion, deferasirox promoted iron depletion-dependent cell death characterized by BclxL downregulation. BclxL overexpression was protective, suggesting a role for BclxL downregulation in iron depletion-induced cell death. This information may be used to develop novel nephroprotective strategies. Furthermore, it supports the concept that monitoring kidney tissue iron depletion may decrease the risk of deferasirox nephrotoxicity.

Deferasirox (also known as ICL670 and marketed as Exjade®, Novartis Pharma AG, Basel, Switzerland) is a potent and specific oral *N*-substituted *bis*-hydroxyphenyl-triazole tridentate iron chelator in clinical use since 2005 as first-line therapy for blood transfusion-related iron overload. Nephrotoxicity is the most serious and common adverse effect^1^. The potential clinical practice extent of the problem has been the subject of alarmist reports published in journals such as The Lancet^2^. In this regard, as of December 2015, deferasirox remained considered as a medicine under additional monitoring status by the European Medicines Agency (EMA) because of its nephrotoxic potential^3^.

Deferasirox nephrotoxicity can result in an acute or chronic decrease in glomerular filtration rate (GFR) and/or features of proximal tubular dysfunction, including Fanconi syndrome (Reviewed in ref. 1). In clinical trials and observational studies, GFR is decreased in 30–100% of patients treated with deferasirox, depending on deferasirox dose, method of assessment of GFR and population studied. However, scarce data are available on the molecular mechanisms of nephrotoxicity and the reasons for the specific proximal tubular sensitivity to the drug. In this regard, the suggestion that nephrotoxicity is related to altered glomerular hemodynamics does not explain clinical manifestations related to proximal tubular injury^1^. Deferasirox has been reported to promote apoptosis of cultured proximal tubular cells, but the molecular triggers and therapeutic implications were not characterized^1^,^4^. *In vivo* deferasirox accumulates in the liver and kidney cortex of rats (Reviewed in ref. 1 Deferasirox readily diffuses into cells but whether transporters increase the entry rate into proximal tubular cells is unknown. Besides higher deferasirox accumulation, proximal tubular cells could be more sensitive to deferasirox toxicity because of their high content of mitochondria, which provide energy for transport processes. Mitochondria are key regulators of intracellular iron homeostasis and key mitochondrial proteins require iron for correct functioning^5^,^6^.

We have now explored the molecular mechanisms of deferasirox nephrotoxicity in cultured proximal tubular cells as well as the opportunities for therapeutic manipulation. Deferasirox-induced proximal tubular cell death had features of both apoptosis and necrosis and was dependent on iron availability. Deferasirox decreased BclxL expression and BclxL overexpression was partially protective. However, other commonly used inhibitors of apoptotic or necroptotic cell death were unable to prevent cell death.

## Results

### Deferasirox induces tubular cell death with features of apoptosis

**Figure 1:**
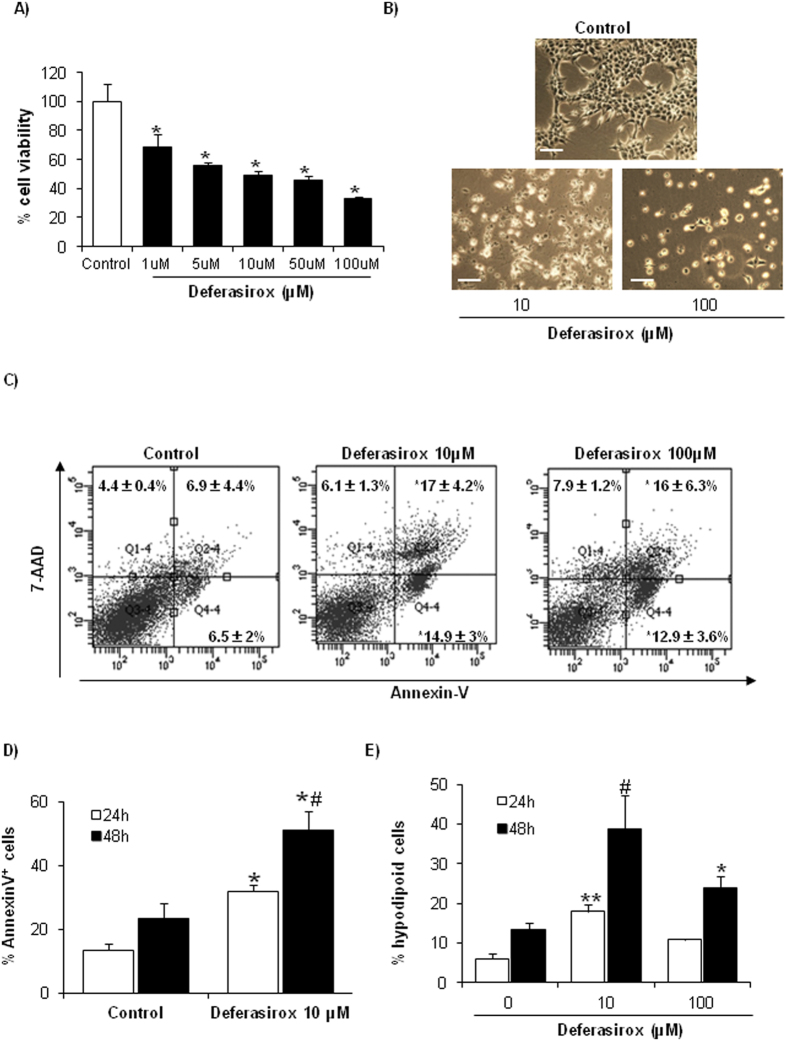
Deferasirox induces death of proximal tubular epithelial cells. Tubular cells were exposed to the indicated concentrations of deferasirox for different times. (**A**) Analysis of tubular cell viability at 24 hours by MTT assay. Mean ± SEM of three independent experiments. *p < 0.001 vs control. (**B**) Contrast phase microscopy photographs (original magnification x200). An example representative of three independent experiments is shown. Note dose-dependent cell detachment. Scale bars: 200 μm. (**C**) Cells were exposed to deferasirox for 24 h, stained with annexin V/7-AAD and analyzed by flow cytometry. Deferasirox increased both annexin V^+^/7-AAD^−^ cells and annexin V^+^/7-AAD^+^ cells. Mean ± SEM of three independent experiments. *p < 0.04 vs control. (**D**) Time-course of annexin V staining in tubular cells exposed to 10 μM deferasirox. Mean ± SEM of three independent experiments. *p < 0.01 vs control; ^#^p < 0.02 vs deferasirox 24 h. (**E**) Hypodiploid cells, suggestive of apoptosis, were assessed by flow cytometry of DNA content. Deferasirox increased the percentage of hypodiploid cells. Mean ± SEM of three independent experiments; *p < 0.0001 vs control 24 h. *p < 0.02 vs control 48 h.

First, the effect of deferasirox on proximal tubular cell viability was tested. Deferasirox decreased tubular cell viability as assessed by the MTT assay (Fig. 1A) and increased cell detachment as assessed by phase contrast imaging (Fig. 1B). Cell death induced by deferasirox is dose-dependent, and the lethal effect is already observed at 1 μM, well within the therapeutic range in humans^3^, although is more evident with higher doses. We used 10 and 100 μM deferasirox to explore the molecular mechanisms of deferasirox nephrotoxicity.

**Figure 2:**
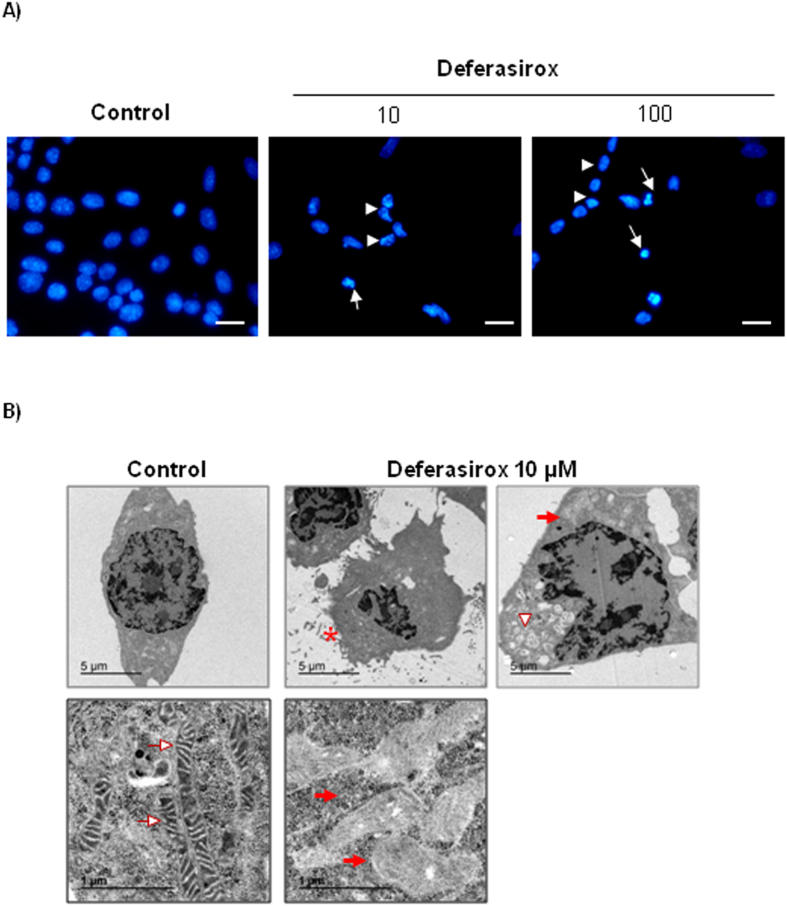
Morphology of proximal tubular cells exposed to deferasirox. (**A**) DAPI-stained cells exposed to deferasirox for 24 hours disclosed both cells with pyknotic, fragmented nuclei suggestive of apoptosis (arrows) and irregular chromatin clumping suggestive of necrosis. (arrowheads). Fluorescence microscopy x200). Scale bars: 50 μm. (**B**) TEM of cells exposed to 10 μM deferasirox for 24 hours disclosed cells with a typical necrotic morphology, characterized by membrane rupture (asterisk), extensive vacuolization (arrowheads), and, loss of mitochondria cristae (red arrows) as compared to normal mitochondria (white arrow) (upper panel ×5000, lower panel ×30000).

Next, features of deferasirox-induced tubular cell death were analyzed in more detail. Cell death was assessed by annexin V/7-AAD staining (Fig. 1C). Deferasirox-induced cell death increased in a time-dependent manner (Fig. 1D).The percentage of hypodiploid cells, typical of apoptosis, was higher at 10 μM than at 50–100 μM deferasirox (Fig. 1E), suggesting a dose-dependent shift in the mode of cell death. Nuclear and cellular morphology was assessed by DAPI staining and transmission electronic microscopy (TEM). DAPI staining disclosed cells with irregular chromatin clumping typical of necrosis as well as cells with pyknotic and fragmented nuclei suggestive of apoptosis (Fig. 2A). TEM identified morphological features typical of necrosis in cells treated with 10 or 100 μM deferasirox, with membrane rupture, irregular chromatin condensation, release of cellular content, damaged mitochondria with loss of cristae (folded structures of the inner membrane), and extensive vacuolization (Fig. 2B).

### Deferasirox induces mitochondrial stress in tubular cells

**Figure 3:**
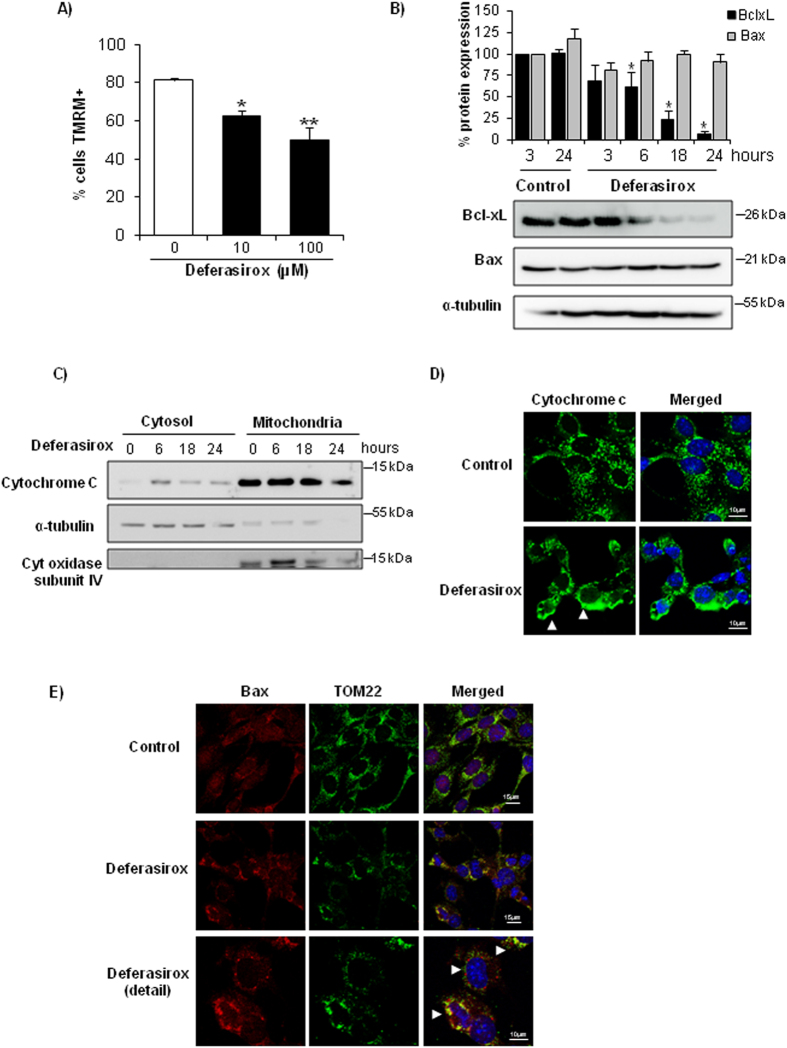
Deferasirox induces mitochondrial stress in tubular cells. (**A**) Tubular cells were exposed to deferasirox for 24 h and loss mitochondrial membrane potential (MMP) was assessed by TMRM staining and flow cytometry. Mean ± SEM of three independent experiments. *p < 0.03 vs control, **p < 0.003 vs control. (**B**) Western blot of BclxL and Bax in tubular cells exposed to 10 μM deferasirox for different times. Data expressed as % change versus control, which was considered to be 100%. Note that BclxL is severely decreased in presence of deferasirox, while Bax does not change, leading to a decreased BclxL/Bax ratio. Mean ± SEM of three independent experiments. *p < 0.003 vs control. Full-length blots is presented in Supplementary Figure 2. (**C**) Cytochrome c is released from mitochondria in tubular cells exposed to deferasirox in a time-dependent fashion. Cytochrome oxidase subunit IV and α-tubulin are controls for fraction separation and loading. Western blot representative of three experiments. Full-length blots is presented in Supplementary Figure 2. (**D**) Localization of cytochrome c in tubular cells exposed to 10 μM deferasirox for 6 hours. Note the punctate mitochondrial pattern in control cells and diffuse labeling in deferasirox-exposed cells (arrowheads). Confocal microscopy. Cytochrome c in green and DAPI in blue, original magnification x63. Images representative of three experiments. (**E**) Deferasirox for 24 hours induces Bax translocation to mitochondria, where it forms aggregates, and colocalizes with mitochondrial receptor TOM22 that appears aggregated (arrowhead). Confocal microscopy: Bax in red, TOM22 in green and DAPI in blue. Original magnification x63, detail x100. Images representative of three experiments.

Mitochondria are involved in different pathways of cell death such as apoptosis and necroptosis^7^,^8^. Furthermore, mitochondria require iron-containing proteins for correct functioning, regulate intracellular iron homeostasis and are very abundant in proximal tubular cells^5^,^6^. Based on this and the morphological evidence of mitochondrial injury, we studied the effect of deferasirox on mitochondria. Deferasirox caused loss of mitochondrial membrane potential as assessed by TMRM staining (Fig. 3A). BclxL and Bax are members of the Bcl2 family of cell death regulatory proteins that act at the mitochondrial level. Since deferasirox induced mitochondrial stress, we measured the BclxL/Bax ratio. Deferasirox downregulated the antiapoptotic protein BclxL while the levels of proapoptotic Bax were unchanged, leading to a decreased BclxL/Bax ratio (Fig. 3B) which is known to predispose to apoptosis^9^. Moreover, cytochrome c was released from mitochondria in a time-dependent manner (Fig. 3C,D). In this line, TOM22, a component of the translocase of the outer mitochondrial membrane (TOM), had a punctate staining pattern corresponding to mitochondrial localization in control cells, while clumping, indicative of loss of mitochondrial integrity, was observed in cells exposed to deferasirox (Fig. 3E). Moreover, in deferasirox-exposed cells, Bax was also clumped and colocalized with TOM22, suggestive of Bax oligomerization at the mitochondrial membrane (Fig. 3E). This is consistent with the role of the TOM complex as a mitochondrial outer membrane receptor required for tBid/Bax binding^10^,^11^,^12^. As mitochondrial stress could result from ROS production, we tested the effect of antioxidants on deferasirox-induced cell death. However, neither the non-specific antioxidant BHA nor the mitochondrial antioxidant Mito-TEMPO offered any protection (Supplementary Figure 1). All together, these results suggest that mitochondria may play a role in deferasirox-induced cell death but this is not mediated by ROS production.

### Deferasirox-induced cell death is not caspase-dependent

**Figure 4:**
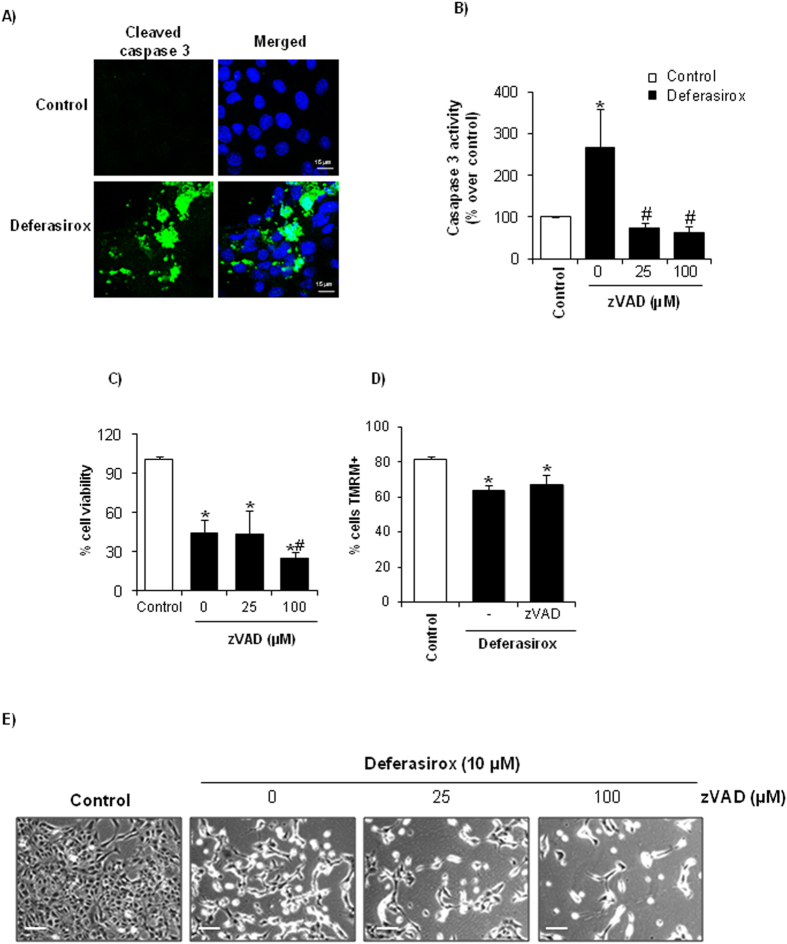
Caspase inhibition prevents caspase-3 activation but does not increase viability of tubular cells exposed to deferasirox. (**A**) Incubation with 10 μM deferasirox for 24 hours resulted in the appearance active caspase-3. Confocal microscopy: cleaved caspase-3 in green and DAPI in blue, original magnification x40. Images representative of three experiments. (**B)** Pretreatment with the pancaspase inhibitor zVAD 1 h before addition of deferasirox prevented caspase-3 activation (activity assay). Mean ± SEM of three independent experiments. *p < 0.03 vs control, ^#^p < 0.001 vs 0 μM zVAD. (**C**) Pretreatment with zVAD does not prevent cell death induced by deferasirox. MTT assay. Mean ± SEM of three independent experiments. *p < 0.003 vs control; ^#^p < 0.04 vs 0 μM zVAD. (**D**) MMP induced by deferasirox is not prevented by 25 μM zVAD, as assessed by flow cytometry of cells stained with TMRM. Mean ± SEM of three independent experiments. *p < 0.003 vs control. (**E**) Contrast phase microscopy photographs showing that zVAD does not prevent deferasirox-induced cell death (original magnification x200, scale bars 100 μm). Note the decreased number of attached cells exposed to deferasirox, which is exacerbated by zVAD. An example representative of three independent experiments is shown.

Release of cytochrome c from mitochondria to cytosol triggers the formation of the apoptosome, activation of caspase 9 and subsequent activation of the caspase cascade leading to activation of the executioner caspase-3 and apoptotic cell death^8^. Thus, we explored caspase activation and its role in deferasirox-induced death. First, we confirmed that deferasirox induced caspase activation since cleaved caspase-3 was observed by confocal microscopy (Fig. 4A). We next studied the effect of caspase inhibition on deferasirox-induced cell death. The broad-spectrum inhibitor of caspases zVAD inhibited deferasirox-induced caspase-3 activation (Fig. 4B). However, zVAD did not prevent cell death induced by deferasirox (Fig. 4C,E). In fact, higher doses of zVAD resulted in increased cell death among deferasirox-treated cells (Fig. 4C,E), as has been previously described for necroptotic cell death^7^,^8^. Moreover, caspase activation did not mediate mitochondrial stress since zVAD did not prevent the loss of mitochondrial membrane potential induced by deferasirox (Fig. 4D).

### Deferasirox-induced cell death is not RIPK1- or RIPK3-dependent

**Figure 5:**
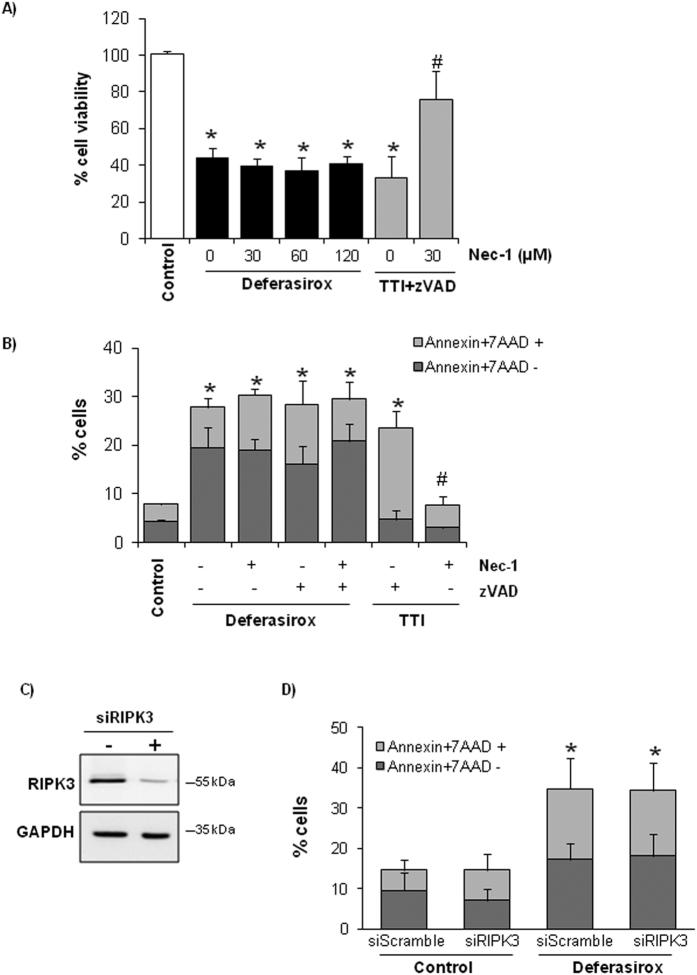
Necroptosis inhibition does not prevent deferasirox-induced tubular cell death. (**A**) Tubular cells were treated with different concentrations of Nec-1 (RIPK1 inhibitor) 1 h before addition of 10 μM deferasirox for 24 h. Nec-1 did not improve cell survival (MTT assay) at any dose. The cytokine cocktail 100 ng/ml TWEAK/30 ng/ml TNFα/30 U/ml interferon-γ (TTI) was used as a positive control for necroptosis and Nec-1. Mean ± SEM of three independent experiments. *p < 0.003 vs control. (**B**) Cells were pretreated with 25 μM zVAD and/or 30 μM Nec-1 before exposure to 10 μM deferasirox for 24 h. Cell death was measured by flow cytometry of annexin V/7-AAD stained cells. Mean ± SEM of three independent experiments. *p < 0.003 vs control. (**C**) RIPK3 expression in tubular cells transfected with a specific siRNA. Representative western blot of three independents experiments. Full-length blot is presented in Supplementary Figure 3. (**D**) RIPK3 was targeted with siRNA, and 48 h later cells were exposed to 10 μM deferasirox for 24 h. Cell death was assessed by flow cytometry of annexin V/7-AAD stained cells. Mean ± SEM of three independent experiments. *p < 0.003 vs control.

Necroptosis is a recently described pathway for cell death, which usually requires RIPK1 and RIPK3 and is inhibited by necrostatin-1 (Nec-1), a RIPK1 inhibitor that is used to distinguish between necroptosis and apoptosis. Since caspase inhibition may favor the occurrence of necroptosis^7^ and caspase inhibition did not prevent or even increased deferasirox-induced tubular cell death (Fig. 4C,E), a potential role for necroptosis in deferasirox nephrotoxicity was functionally explored. However, Nec-1 did not protect from deferasirox-induced cell death, assessed either as cell viability (MTT assays) or as annexin V/7-AAD staining (Fig. 5A,B). Since, apoptosis and necroptosis may coexist, cells were pre-treated with Nec-1 and zVAD before deferasirox, but this combination did not decrease cell death either (Fig. 5B). Moreover, RIPK3 knockdown with siRNA did not decrease deferasirox-induced cell death (Fig. 5C,D). These experiments were also performed with higher concentrations of deferasirox (100 μM) and similar results were obtained (data not shown). Thus, deferasirox does not engage the RIPK1 or RIPK3 necroptosis pathways. The cytokine cocktail TNF-α/TWEAK/Interferon-γ (TTI) + zVAD was used as a positive control for Nec-1-responsive necroptosis^7^,^8^.

### Overexpression of BclxL partially protects tubular epithelial cells against deferasirox-induced cell death

**Figure 6:**
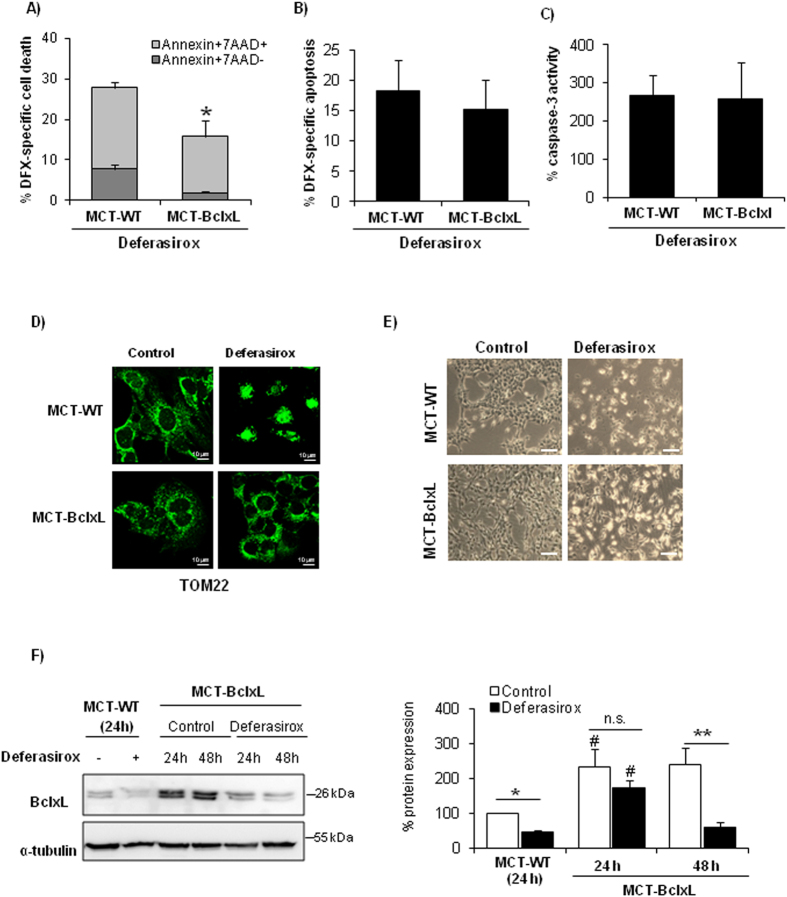
BclxL downregulation is involved in deferasirox-induced cell death. Deferasirox toxicity was explored in BclxL-overexpressing MCT tubular cells (MCT-BclxL) and wild type cells (WT-MCT). (**A)** BclxL-overexpressing cells were protected from cell death induced by exposure to 10 μM deferasirox for 24 h, as assessed by flow cytometry of annexin V/7-AAD stained cells. Mean ± SEM of four independent experiments. *p < 0.03 vs control WT cells. (**B**) BclxL overexpression did not significantly decrease the presence of hypodiploid cells. Mean ± SEM of three independent experiments. (**C**) BclxL overexpression did not prevent caspase-3 activation induced by deferasirox. Mean ± SEM of three independent experiments. (**D)** TOM22 staining. MCT-BclxL cells, but not WT cells, preserve mitochondrial integrity in presence of deferasirox. Representative images of three independents experiments. Magnification x63. (**E**) Contrast phase microscopy photographs of MCT-WT and MCT-BclxL (original magnification x200, scale bars 200 μm). An increased number of cells that remain attached to the plate is observed in MCT-BclxL cells exposed to deferasirox. Representative images of three experiments. (**F**) BclxL protein expression in MCT-BclxL cells during exposure to deferasirox assessed by western blot. Mean ± SEM of three independent experiments. *p < 0.05 vs control MCT-WT; **p < 0.005 vs control MCT-BclxL; ^#^p < 0.004 vs MCT-WT. Full-length blot is presented in Supplementary Figure 3.

BclxL protects cells from a wide variety of lethal stimuli, and BclxL binding to Bax antagonizes its deleterious effect over mitochondria^13^. The BclxL/Bax ratio determines cell sensitivity to lethal stimuli^14^,^15^. In this sense, BclxL protects from paracetamol nephrotoxicity, another caspase-independent form of nephrotoxic cell death^16^. Therefore, we tested the role of BclxL in deferasirox-induced cell death using tubular cells stably overexpressing BclxL. BclxL overexpression partially protected from cell death induced by deferasirox as assessed by annexin V/7-AAD staining (Fig. 6A). However, BclxL overexpression did not significantly reduce the number of hypodiploid cells nor prevented caspase-3 activation, indicating that BclxL downregulation contributes to non-apoptotic cell death induced by deferasirox (Fig. 6B,C). Moreover, BclxL overexpression preserved mitochondrial integrity as assessed by TOM22 staining (Fig. 6D). Morphological studies confirmed that protection offered by BclxL overexpression is partial (Fig. 6E). This result suggests that BclxL overexpression partially protects from deferasirox toxicity. Partial protection may result from higher basal levels of BclxL protein in BclxL overexpressing, leading to a delay in the reduction of BclxL protein levels below a critical threshold in response to deferasirox, as compared to wild-type cells (Fig. 6E).

### Deferasirox-induced cell death results from iron depletion

**Figure 7:**
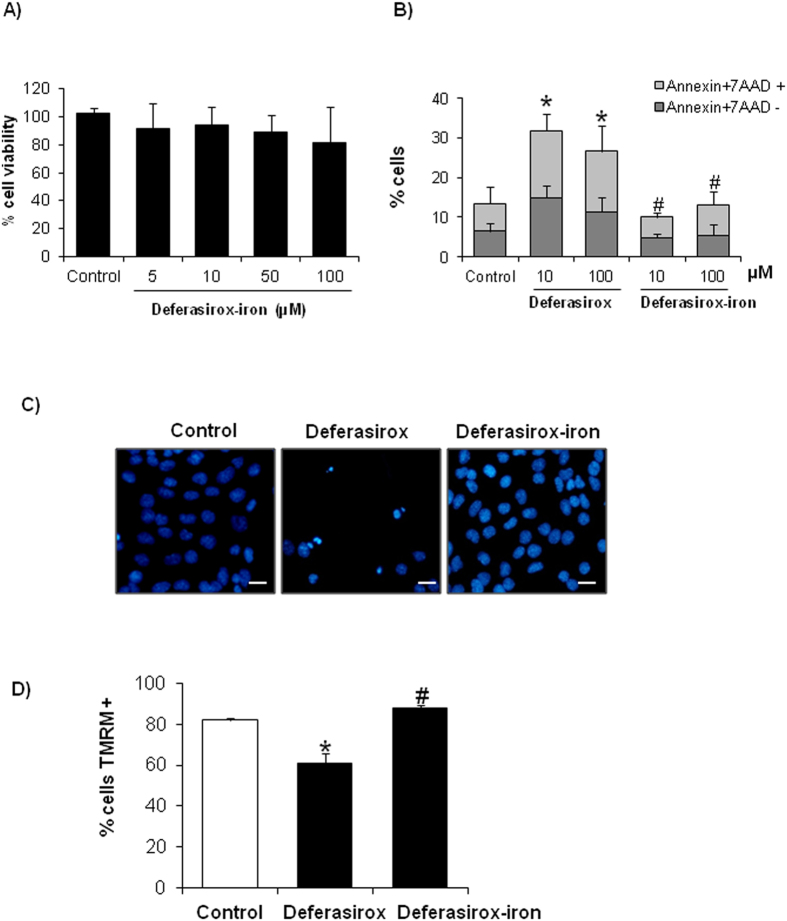
Deferasirox-iron complex does not induce tubular cell death. Tubular cells were exposed to different concentrations of deferasirox-iron complex for 24 h. (**A**) Viability was assessed by MTT assay. Deferasirox-iron complex was not toxic in tubular cells. Mean ± SEM of four independent experiments. (**B**) Cell death was assessed by annexin V/7-AAD staining and flow cytometry in tubular cells treated with deferasirox or deferasirox-iron complex. Mean ± SEM of four independent experiments. *p < 0.03 versus control, ^#^p < 0.001 vs deferasirox. (**C)** Tubular cells treated with deferasirox-iron complex do not present pyknotic nuclei and ramain attached to the plate. DAPI staining. Magnification x200. Scale bars: 50 μm. (**D**) Deferasirox-iron complex does not induce MMP loss in tubular cells. Graph shows TMRM staining assessed by flow cytometry. Mean ± SEM of four independent experiments. *p < 0.03 versus control, ^#^p < 0.001 vs deferasirox.

**Figure 8:**
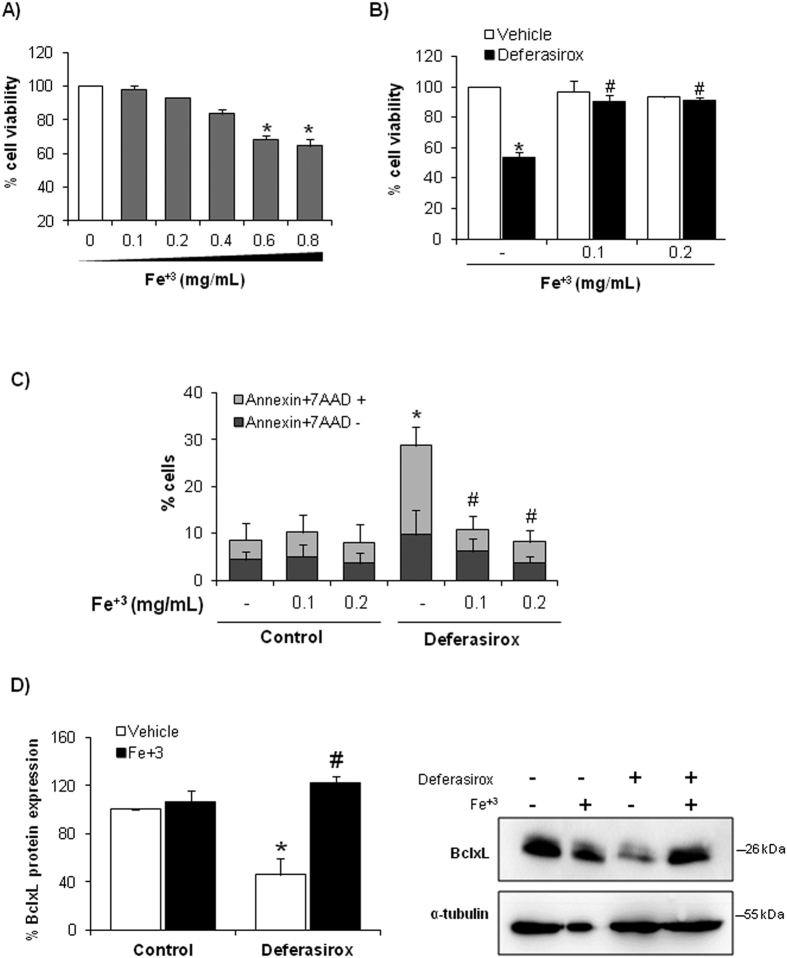
Iron loading prevented deferasirox-induced tubular cell death. (**A)** Cell viability assay (MTT) in tubular cells treated with different concentrations of iron citrate for 24 h. Mean ± SEM of four independent experiments. Non-toxic concentrations were chosen for further experiments. (**B**) Tubular cells were cultured with 0.1 or 0.2 mg/mL iron citrate before exposure to 10 μM deferasirox for 24 h. Deferasirox toxicity is not observed in cells pre-incubated with iron citrate. MTT assay. Mean ± SEM of four independent experiments. *p < 0.001 vs control; ^#^p < 0.001 vs deferasirox alone. (**C**) Iron loading prevented cell death induced by deferasirox. Flow cytometry of annexin V/7-AAD staining. Mean ± SEM of four independent experiments. *p < 0.001 vs control; ^#^p < 0.001 vs deferasirox alone. (**D**) BclxL expression is preserved in MCT cells overloaded with iron and treated with deferasirox. Mean ± SEM of three independent experiments; *p < 0.001 vs vehicle control; ^#^p < 0.0001 vs Deferasirox alone. Full-length blot is presented in Supplementary Figure 3.

We then focused on whether deferasirox nephrotoxicity is dependent on iron depletion or whether it might result from iron depletion-independent, off-target effects of the drug. For this, tubular cells were treated with deferasirox-iron complex, in which deferasirox is saturated with iron before addition to the cell culture. Deferasirox-iron complex did not deplete cellular iron and was not toxic to tubular cells as assessed by either cell viability assays (Fig. 7A), annexin V/7-AAD staining (Fig. 7B), nuclear morphology (Fig. 7C) or MMP loss (Fig. 7D). To corroborate the protective effect of iron repletion, we studied the effect of deferasirox in tubular cells loaded with iron. First, we search for a non-toxic dose of iron. We used no toxic dose of iron (0.1–0.2 mg/ml) to study the effect of iron over deferasirox-induced cell death (Fig. 8A). Subsequently, tubular cells were loaded with 0.1 or 0.2 mg/ml iron citrate for 1 h before exposure to deferasirox for 24 h. Iron-loaded tubular cells were protected from deferasirox lethal effect as assessed by viability assays and annexin V/7-AAD staining (Fig. 8B,C). Iron loading prevented BclxL downregulation (Fig. 8D), indicating that iron depletion drives deferasirox-induced BclxL downregulation and cell death.

Ferroptosis is a recently described pathway for cell death, which requires iron and is inhibited by ferrostatin-1^17^. As both, ferroptosis and deferasirox-induced cell death, are dependent on intracellular iron levels, we explored whether these cell death pathways were related. We ruled out a role of ferroptosis in deferasirox-induced cell death since pre-treating the cells with ferrostatin-1 was not protective (Supplementary Figure 1C). This is coherent with the need of iron for ferroptosis.

## Discussion

Nephrotoxicity is the dose-limiting side effect of deferasirox. Some deferasirox users, such as children with transfusion-dependent anemia, may require deferasirox for decades, while elderly patients with myelodysplastic syndromes may be more sensitive to nephrotoxicity because of aging kidneys. Thus, unraveling the molecular and cellular mechanisms of nephrotoxicity may allow the development of less toxic analogues or nephroprotective strategies. The key findings of the present study are that (i) at clinically relevant concentrations, deferasirox induces proximal tubular cell death with features of apoptosis and necrosis, but not responsive to caspase inhibition or RIPK targeting. However, deferasirox decreased BclxL and BclxL overexpression protected from deferasirox-induced cell death. (ii) Deferasirox-induced BclxL downregulation and nephrotoxicity were totally dependent on iron depletion. Thus, deferasirox may serve as a model of iron-depletion-induced cell death and monitoring kidney cell iron content may help prevent deferasirox nephrotoxicity.

The mechanisms of deferasirox nephrotoxicity are unclear. A single peer-reviewed report has explored the cellular mechanism of deferasirox nephrotoxicity. In rats, deferasirox increased urinary protein and glucose excretion, suggesting proximal tubular injury, despite normal glomerular filtration^4^. In addition, deferasirox induced apoptosis of cultured tubular cells, suggesting that proximal tubular cell injury is a key feature of nephrotoxicity^4^. However, therapeutic manipulation of deferasirox nephrotoxicity was not attempted. Additional information available at Food and Drug Administration and European Medicines Agency websites as part of the file on the drug submitted for regulatory approval (http://www.ema.europa.eu; http://www.fda.gov/). However, this information was not peer-reviewed. Thus, the manufacturer hypothesized that modulation of hemodynamics by excessively rapid iron removal was the mechanism for nephrotoxicity^18^. However, a hemodynamically mediated decrease in glomerular filtration does not cause Fanconi syndrome. Sublethal or lethal tubular cell injury might explain Fanconi syndrome, the lack of return to baseline renal function or the lag time of days to return to normal, and the severe deterioration of renal function requiring dialysis that have been described in patients on deferasirox^1^. Our results confirm that deferasirox is directly toxic to proximal tubular cells and point to iron depletion as the major underlying mechanism. In this regard, they support the concept hypothesized by the makers of deferasirox to the European Medicines Agency, that overchelation may contribute to nephrotoxicity^18^, although the consequence of overcorrection appears to be direct cellular toxicity. Clinical trials support the clinical relevance of our findings, since increases in serum creatinine were observed most frequently in the population of patients having the most dramatic decrease in liver iron and serum ferritin, although specific figures were not provided^19^. Intracellular iron is necessary for oxidative phosphorylation and ATP production and might be reduced to critically low levels after deferasirox therapy in cells that are not overloaded with iron to start with or which accumulate deferasirox intracellularly. Consistently, in clinical trials deferasirox nephrotoxicity developed in patients with the greatest reduction in iron burden^1^. However, kidney injury is not a classical manifestation of iron deficiency, and iron chelators, such as deferoxamine prevent experimental kidney injury^20^,^21^,^22^. Indeed, a form of iron-dependent cell death, ferroptosis, has been observed in kidney cells^23^,^24^,^25^. However, in cultured non-renal cells iron chelation by deferoxamine was deleterious, and promoted cell senescence, decreased mitochondrial respiration through a defective complex II activity and decreased expression of the iron–sulfur protein subunit^26^. Although we did not explore cell senescence, our results are in accordance to this observation, since iron depletion in tubular cell was also deleterious and related to mitochondrial changes. It is likely that below a critical level, iron depletion results in cell toxicity.

Deferasirox-induced tubular death had features of both apoptosis and necrosis and there was morphological evidence of mitochondrial injury. However, cell death could not be prevented by targeting caspases, as would be expected for apoptosis or RIPK1/RIPK3 as would be expected for necroptosis. BclxL is a negative regulator of tubular cell death in AKI and BclxL overexpression protects against paracetamol nephrotoxicity, another form of tubular cell death not responsive to caspase inhibitors^16^. BclxL can sequester proapoptotic molecules such as Bax, protects mitochondria from injury induced by different factors, including processed Bid or hydrogen peroxide^27^,^28^ and has long been known to protect from both necrosis and apoptosis, as in the case of hypoxia (without reoxygenation)-induced death or from chemical hypoxia such as that induced by cyanide. Cyanide-induced cell death is characterized by inhibition of the respiratory chain reaction and necrotic features including remarkable mitochondrial swelling with loss of cristae and loss of plasma membrane integrity^29^,^30^. It is possible that BclxL is additionally protective by increasing mitochondrial efficiency through a direct interaction with the β-subunit of the F(1)F(O) ATP synthase, decreasing an ion leak within the F(1)F(O) ATPase complex^31^. In this regard, some cyanide-induced death features are similar to deferasirox-induced death, which may also interfere with the function of iron-containing respiratory chain proteins. Deferasirox decreased BclxL levels in both wild type and BclxL overexpressing cells suggesting an effect distal to gene transcription since the regulation of gene transcription differs between the two cell types. Thus, BclxL may be a target of proteases and BclxL degradation accelerates cell death^32^. Progressive, deferasirox-induced reductions in BclxL expression also in BclxL overexpressing cells may explain the observation of partial protection from deferasirox-induced death. A decreased BclxL expression had been previously observed in another model of iron depletion: the use of anti-transferrin receptor 1 (TfR1, CD71) antibodies in malignant cells to reduce cell surface TfR1 expression. This caused iron depletion, decreased BclxL expression and promoted cell death and primed for cell death induced by chemotherapeutic agents^33^. The TOM complex is a receptor of the mitochondrial outer membrane required for tBid/Bax-induced cytochrome c release^10^. Specifically, TOM22, a member of TOM complex, is a mitochondrial receptor for Bax that could modulate the mitochondrial translocation of Bax^11^,^12^. In this regard, we observed mitochondrial translocation of Bax and colocalization in clumps with TOM22 in deferasirox-treated cells. This fits well with the our observation of protection by BclxL and the well-known protective effect of BclxL against Bax-induced death^34^.

Proximal tubular cells are designed for heavy transmembrane transport and are rich in mitochondria that provide energy for transport processes, and in membrane transporters that contribute to excretion of organic anions and cations and to the recovery of huge amounts of nutrients and metabolites from the glomerular ultrafiltrate. Mitochondrial dysfunction could limit these transport processes and result in manifestations of proximal tubular dysfunction (Fanconi syndrome) even in the absence of cell death. In this regard, the deferasirox concentration used in the present study to define molecular mechanisms and response to interventions is a clinically attainable deferasirox concentration. Thus, circulating Cmax for deferasirox after a single oral dose of 20 mg/kg, hovers around 100 μM^35^. In this regard, deferasirox was already toxic at 1 μM in cultured cells, well within the therapeutic concentration range.

It may appear contradictory that deferasirox is used in conditions of iron overload yet nephrotoxicity may be related to iron deficiency. Two facts may explain this apparent contradiction. First, following repetitive blood transfusion, iron overload takes place mainly in the liver and heart, and deferasirox therapy is aimed at clearing these two organs^36^. Whether proximal tubular cells are iron overloaded at baseline and their degree of iron clearance is not factored in into clinical decision-making. However, in preclinical studies, the liver and the kidney cortex were the organs that accumulated more deferasirox^18^. The combination of lower initial baseline iron deposits with a higher local deferasirox accumulation may facilitate the development of nephrotoxicity due to excessive iron depletion, even when liver or heart deposits have not yet been cleared. Furthermore, a recent Mendelian randomization study of the effect of serum iron levels on eGFR, found a 1.3% increase in eGFR per standard deviation increase in iron and the results for ferritin were consistent with those for iron. This study suggested for the first time a protective effect of iron on kidney function in the general population^37^. On a more basic level, iron was identified as a key regulator of mitochondrial biogenesis. Depletion of cellular iron resulted in a rapid, dose-dependent decrease of select mitochondrial protein levels and oxidative capacity across a broad range of cell types and fully reversed when iron is reintroduced^38^. In this regard, both inadequate and excessive iron causes significant mitochondrial malfunction, mtDNA damage and oxidative stress in rats, including kidney oxidative stress^39^,^40^. Thus, our studies may help advance in the understanding of the deleterious effect of iron deficiency on the kidney. Iron deficiency was one of the eight causes of chronic disease and injury that affected more than 10% of the world population in 2015^41^.

**Figure 9:**
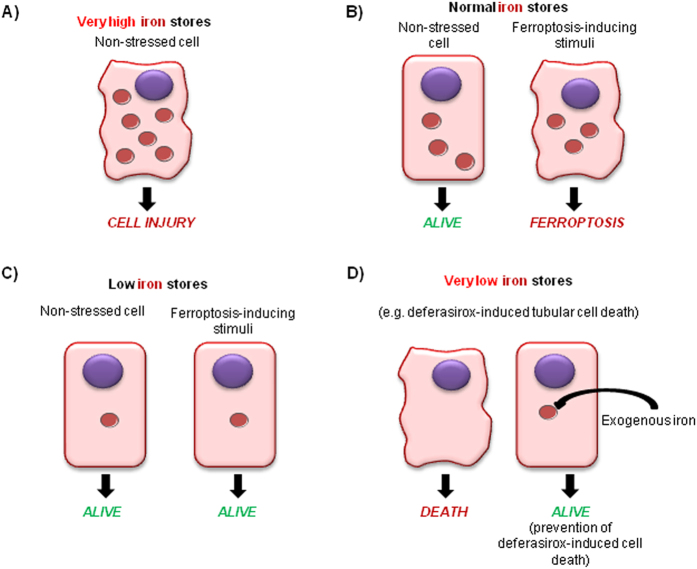
The spectrum of cell injury dependent on derangements of cellular iron contents. (**A**) Excess tubular cell iron loading, as in the context of hemoglobinuria or myoglobinuria, may cause tubular cell injury. (**B**) Normal cell iron content is required for physiological cell function, but in the presence of ferroptosis inducers, it facilitates the occurrence of this iron-catalyzed cell death. (**C**) Mild iron depletion that still allows physiological cell function protects cells from death induced by ferroptosis inducers. (**D**) Severe iron depletion, as induced by deferasirox, is incompatible with physiological cell function and promotes cell death. This form of cell death associated with severe iron depletion may be rescued by a low, non-toxic iron supplementation.

Ferroptosis is a recently recognized form of iron-catalyzed regulated necrosis^42^,^43^,^44^, which was recently shown to play a key role in acute kidney injury^23^,^24^. In this regard, our data complete the spectrum of cell injury dependent on derangements of cellular iron contents (Fig. 9). Excess tubular cell iron loading, as in the context of hemoglobinuria or myoglobinuria, may cause tubular cell injury^45^,^46^. Normal cell iron content is required for physiological cell function, but in the presence of ferroptosis inducers, it facilitates the occurrence of this iron-catalyzed cell death. Iron depletion that still allows physiological cell function, protects cells from ferroptosis inducers. The data presented in the present manuscript belong to a more severe iron depletion, which is incompatible with physiological cell function and promotes cell death.

In conclusion, deferasirox is directly toxic to cultured proximal tubular cells and induces mitochondrial dysfunction and cell death with mixed features of apoptosis and necrosis. Toxicity is intrinsic to the mechanism of action of the drug since it depends on iron depletion-induced downregulation of BclxL and does not appear to be an off-target effect. This observation supports the concept that a less aggressive iron depletion strategy and withholding the drug when serum ferritin approaches physiological levels may decrease the risk of deferasirox nephrotoxicity. In current clinical practice close monitoring of iron stores may be used to decrease the risk of toxicity by decreasing or stopping the drug once iron deposits fall or at the earliest sign of kidney injury. However, developing methods for specific monitoring of kidney iron stores may help to further limit nephrotoxicity. Further drug structural modifications to limit toxicity may be aimed at preventing proximal tubular cell loading. Deferasirox-induced mitochondrial dysfunction and death cannot be rescued by commonly used apoptosis and necroptosis inhibitors. However, BclxL was protective, further pointing out to mitochondria as sites of injury. Thus, deferasirox provides a new and challenging model of proximal tubular cell death that may provide new clues to the molecular mechanisms of nephrotoxicity and to the adverse consequences of iron depletion.

## Methods

### Cells and reagents

MCT cells are a proximal tubular epithelial cell line harvested originally from the renal cortex of SJL mice and have been extensively characterized^47^. They were cultured in RPMI 1640 (GIBCO, Grand Island, NY), 10% decomplemented fetal bovine serum (FBS), 2 mM glutamine, 100 U/mL penicillin and 100 μg/mL streptomycin, in 5% CO_2_ at 37° 48. MCT cells overexpressing BclxL has been previously described^49^,^50^. Cells were seeded in 10% FCS RPMI overnight, and then rested in serum-free medium for 24 h. Thereafter, stimuli were added to subconfluent cells.

Deferasirox and deferasirox-iron complex (Santa Cruz Biotechnology, Santa Cruz, CA) were dissolved in DMSO and methanol respectively. Benzyloxycarbonyl-Val-Ala-DL-Asp-fluoromethylketone (zVAD-fmk) (BD Bioscience, San Jose, CA, USA), ferrostatin-1 (Santa cruz Biotechnology), necrostatin-1 (Sigma, St. Louis, MO, USA) was dissolved in DMSO and added 1 hour prior to stimuli. The concentration of zVAD-fmk (25–100 μM), necrostatin-1 (30–120 μM) and ferrostatin-1 (1–10 μM) was based on prior experience and dose-response studies performed in the lab and shown to protect MCT cells from apoptosis- and necroptosis-inducing stimuli^7^,^51^. The antioxidant butylated hydroxyanisole (BHA)(Sigma) was used at 100 μM, based on prior experience in the lab in preventing necroptosis-induced cell death in the same cell system^8^, while Mito-TEMPO (Axxora, New Yor, NY) was used at several concentrations^52^,^53^. The cytokine cocktail 100 ng/ml TWEAK/30 ng/ml TNFα/30 U/ml interferon-γ (TTI) was used as a positive control for necrostatin-1-responsive necroptosis^7^,^8^. Ammonium iron (III) citrate-reagent (Sigma) was dissolved in bidistilled water. The concentration of iron citrate was derived from the literature^54^.

### Assessment of cell death

Cell viability was estimated using the 3-[4,5-dimethylthiazol-2-yI]-2,5 diphenyltetrazolium bromide (MTT, Sigma) colorimetric assay. Following stimulation, culture medium was removed, and cells were incubated with 1 mg/mL MTT in PBS for 1 h at 37 °C. The resulting formazan crystals were dried and dissolved in DMSO. Absorbance (indicative of cell viability) was measured at 570 nm.

To assess hypodiploid cells, 10,000 cells were seeded in 12-well plates (Costar, Cambridge, MA). Following stimulation, adherent cells were pooled with spontaneously detached cells, and incubated in 100 μg/mL propidium iodide (PI), 0.05% NP-40, 10 μg/mL RNAse A in PBS at 4 °C for >1 h. This assay permeabilizes the cells, thus PI stains both live and dead cells. The percentage of apoptotic cells with decreased DNA staining (hypodiploid cells) was counted by flow cytometry using FACS Canto cytometer and FACS Diva Software (BD Biosciences)^8^.

For assessment of cell death by annexin V/7-amino-actinomycin D (7-AAD) staining, 5 × 10^5^ cells were washed with ice-cold PBS, resuspended in 100 μl binding buffer, and stained with 2.5 μl PE-Annexin V and 5 μl 7-AAD. Cells were incubated for 15 min at 37 °C in the dark. Then, 400 μl binding buffer was added just before flow cytometry. Cells were analyzed using FACS Canto cytometer and FACS Diva Software (BD Biosciences). Early and late cell death was evaluated on PE fluorescence (Annexin V) versus PerCP (7-AAD) plots. Cells stained only with annexin V were evaluated as being in early cell death; cells stained with both annexin V and 7-AAD were evaluated as being in late cell death^7^.

Nuclear morphology was assessed in formalin-fixed cells stained with DAPI (Sigma) and observed with fluorescence microscopy (Nikon E600). Cell morphology was further examined by transmission electron microscopy. Cells were fixed in 4% formaldehyde 2% glutaraldehyde in PBS, dehydrated, embedded in Epon resin, cut, and observed in a Jeol Jem1010 (100 Kv) microscope.

### Mitochondrial membrane potential (MMP)

Changes in MMP were determined as differences in tetramethylrhodamine methyl ester (TMRM) fluorescence (Molecular Probes, Thermo-Fisher Eugene, OR). Adherent cells were pooled with spontaneously detached cells and stained with 150 nM TMRM for 10 min at 37 °C. Fluorescence intensity was measured by flow cytometry using FACS Canto cytometer and FACS Diva Software (BD Biosciences). Decreased TMRM fluorescence indicates reduced MMP.

### Caspase 3 activity

Caspase-3 activity (MBL, Nagoya, Japan) was measured following the manufacturer’s instructions^8^. In brief, cell extracts (70 μg protein) were incubated with 200 μM DEVD-pNA and pNA light emission was quantified. Comparing the pNA absorbance of an apoptotic sample with a control allows determination of the fold increase in caspase activity.

### Immunofluorescence

Cells plated onto Labtek™ slides were fixed in 4% paraformaldehyde and permeabilized in 0.2% Triton X-100/PBS, washed in PBS and incubated with anti-cytochrome c (1:500, BD Pharmigen), anti-Bax (1:50, Santa Cruz) or anti-TOM22 (1:50, Abcam) followed by Alexa488 or Alexa562 conjugated secondary antibody respectively (1:300, Invitrogen)^55^. Nuclei were counterstained with DAPI.

### Western blot

Cell samples were homogenized in lysis buffer (50 mM TrisHCl, 150 mM NaCl, 2 mM EDTA, 2 mM EGTA, 0.2% Triton X-100, 0.3% NP-40, 0.1 mM PMSF and 1 μg/ml pepstatin A) then separated by 10% SDS-PAGE under reducing conditions. After electrophoresis, samples were transferred to PVDF membranes (Millipore), blocked with 5% skimmed milk in PBS/0.5% v/v Tween 20 for 1 hour, washed with PBS/Tween, and incubated with anti-Bax (1:100, Calbiochem), anti-BclxL (1:250, Santa Cruz Biotechnology) or anti-RIPK3 (1:1000, Novus Biologicals, Littleton, CO) diluted in 5% milk PBS/Tween. Blots were washed with PBS/Tween and incubated with appropriate horseradish peroxidase-conjugated secondary antibody (1:5000, GE Healthcare). After washing with PBS/Tween, blots were developed with the chemiluminescence method (ECL) (Millipore) and probed with anti-α-tubulin antibody (1:10,000, Sigma) or anti-GAPDH (1:5,000, Millipore). Levels of expression were corrected for minor differences in loading.

The release of cytochrome c from mitochondria to cytosol was also assessed by Western blot. Mitochondria-free cytosolic extracts or mitochondria were electrophoresed on 15% polyacrylamide gels and analyzed by Western blot^8^. Anti-cytochrome c (1:500, Santa Cruz Biotechnology) was used. Cytochrome oxidase subunit IV (1:500, Molecular Probes, Leiden, The Netherlands) is not released from mitochondria during apoptosis, and was used as control.

### siRNA transfection

Cells were seeded at in 6-well plates and transfected on the following day with 20 nM scrambled siRNA or siRNA against RIPK3 (Invitrogen) by using lipofectamine (Invitrogen, Thermo-Fisher)^56^. After 48 h, the transfected cells were treated with deferasirox for 24 h and harvested for flow cytometry analysis of cell death.

### Statistics

Statistical analysis was performed using SPSS 11.0 statistical software. Results are expressed as mean ± SEM. Significance at the p < 0.05 level was assessed by Student´s t test for two groups of data and ANOVA for three of more groups.

## Additional Information

**How to cite this article**: Martin-Sanchez, D. *et al*. Deferasirox-induced iron depletion promotes BclxL downregulation and death of proximal tubular cells. *Sci. Rep.* **7**, 41510; doi: 10.1038/srep41510 (2017).

**Publisher's note:** Springer Nature remains neutral with regard to jurisdictional claims in published maps and institutional affiliations.

## Supplementary Material

Supplementary Figures
