# Supplementary material for: Deferasirox-induced iron depletion promotes BclxL downregulation and death of proximal tubular cells
Source: Sci Rep. 2017 Jan 31;7:41510. doi: 10.1038/srep41510 (PMC5282523; doi:10.1038/srep41510)

Deferasirox-induced iron depletion promotes BclxL downregulation and death of proximal tubular cells

Diego Martin-Sanchez[1-3], Angel Gallegos-Villalobos[1-3], Miguel Fontecha-Barriuso[1-3], Susana Carrasco[1-3], Maria Dolores Sanchez-Niño[1-3], Francisco J Lopez-Hernandez[2-4], Marta Ruiz-Ortega[1-3], Jesus Egido[1,2], Alberto Ortiz[1-3], Ana Belén Sanz[1-3].

Supplementary figure 1

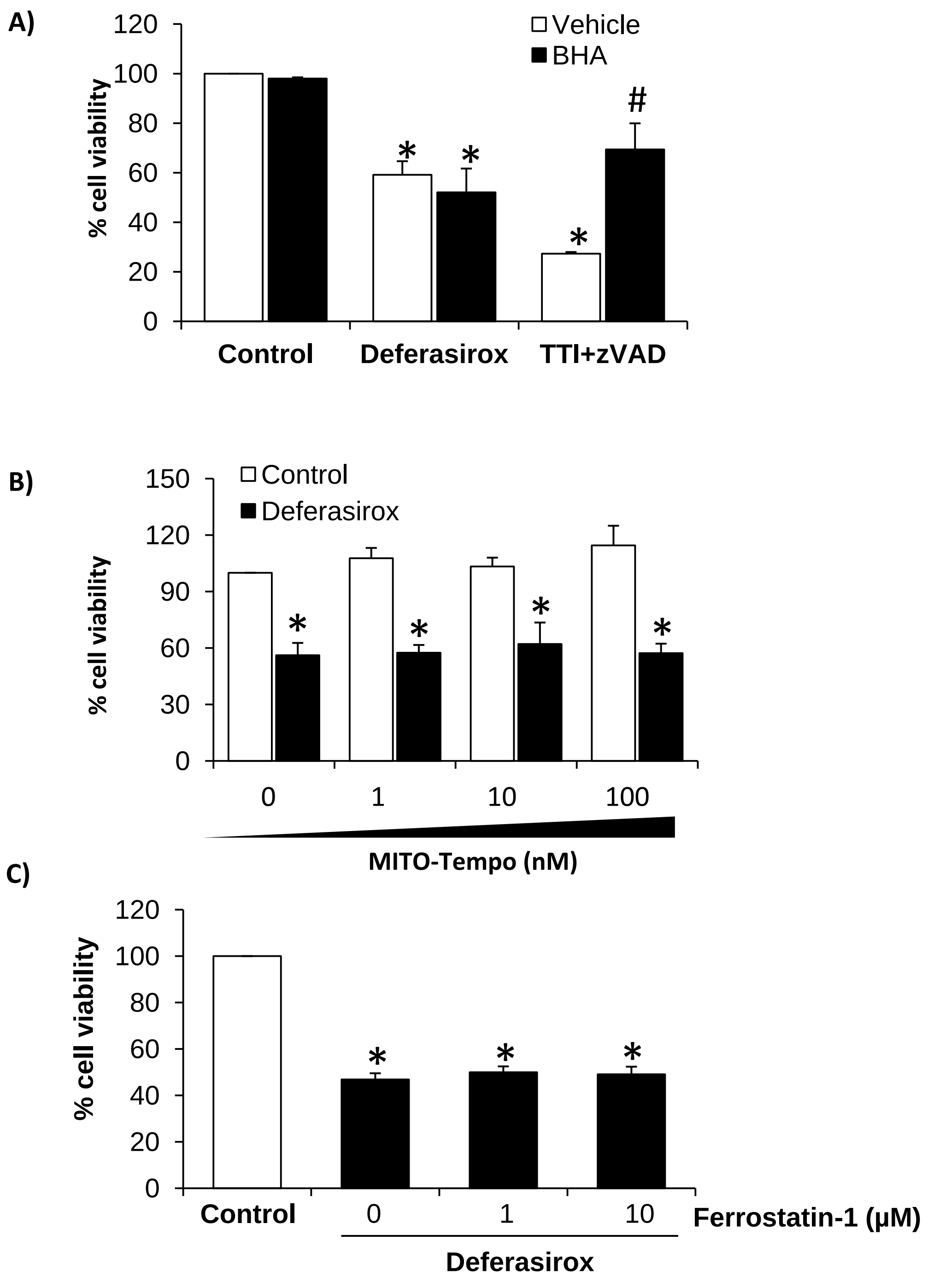

Supplementary figure 1. Antioxidants or ferrostatin-1 did not prevent deferasirox-induced cell death in tubular cells. Tubular cells were treated with butylated hydroxyanisole (BHA) at 100 µM **(A)**, with Mito-TEMPO at different concentrations (**B**), or with Ferrostatin-1 at different concentrations (**C**), for 1 hour and then they were incubated with 10 µM deferasirox for 24 hours. Cell death was assessed by MTT assay. The cytokine cocktail 100 ng/ml TWEAK/30 ng/ml TNFα/ 30 U/ml interferon-γ (TTI)+zVAD was used as a positive control for BHA protection [13]. Mean ± SEM of three independent experiments.*$p<0.02$ vs control; #$p<0.04$ vs TTI+zVAD.

Supplementary Figure 3. Image of uncropped blots

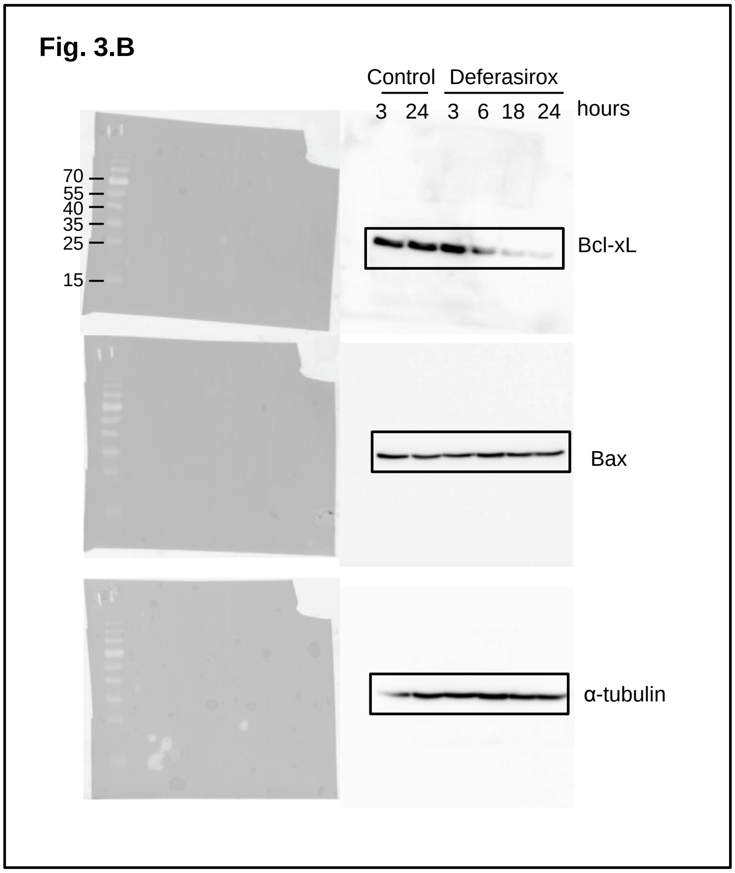

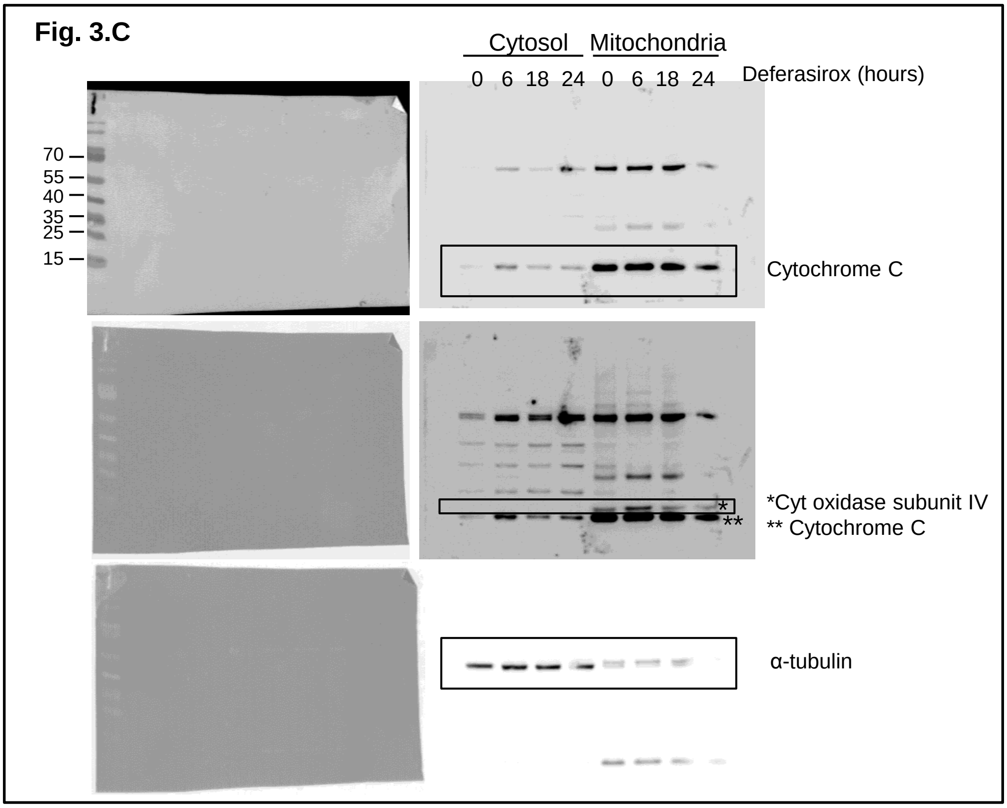

Supplementary Figure 4. Image of uncropped blots part II

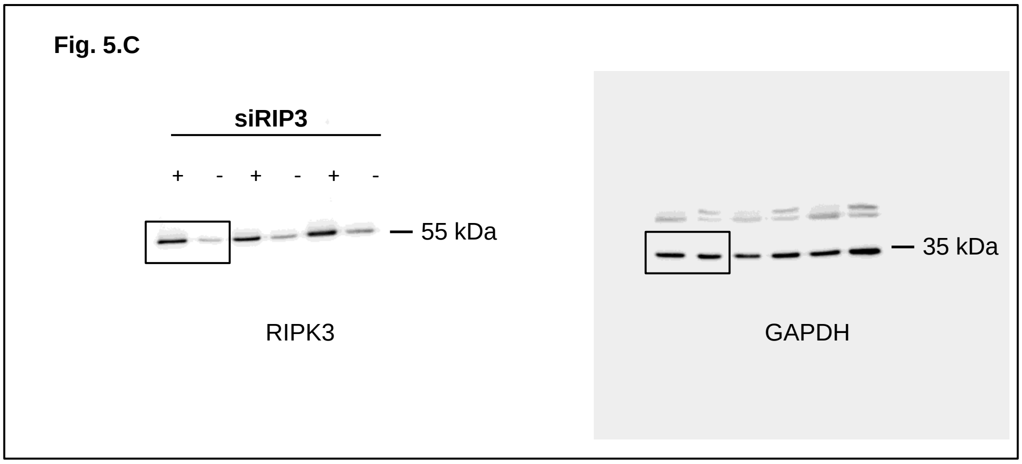

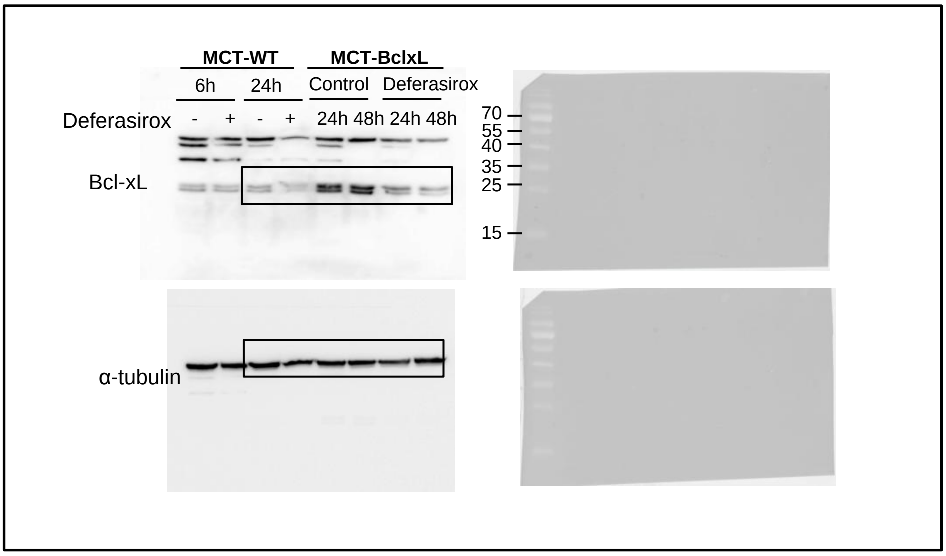

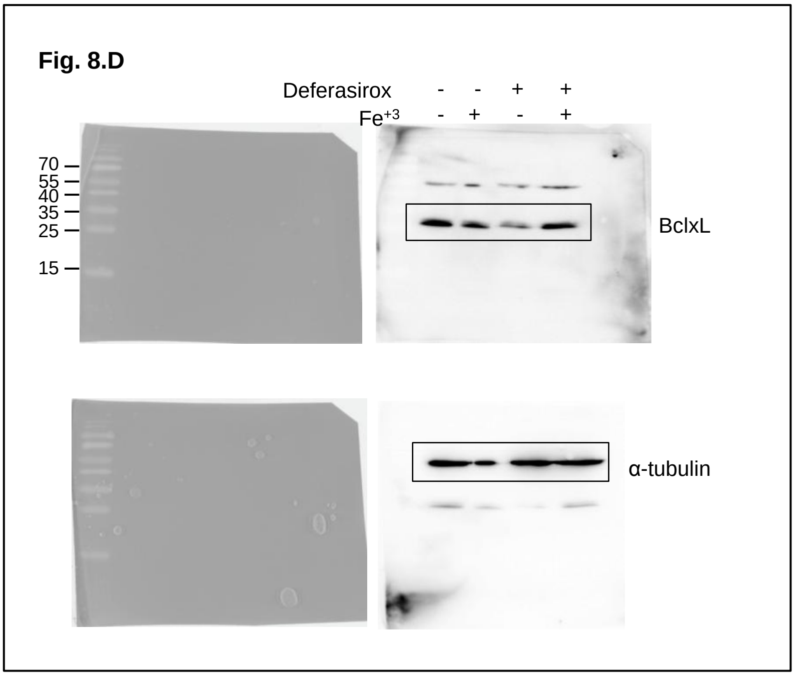

Supplement: Supplementary Figures [file srep41510-s1.pdf]
